# The Importance of Redefining Geriatric Expertise in Nursing Homes to Reduce Unnecessary Hospitalizations

**DOI:** 10.1093/geroni/igab046.2121

**Published:** 2021-12-17

**Authors:** Franziska Zúñiga, Lori Popejoy, Amy Vogelsmeier

## Abstract

Unplanned transfers from nursing homes (NHs) are burdensome, associated with adverse outcomes for residents and costly for health care systems. Internationally, NHs are facing similar issues whereby a lack of geriatric expertise combined with a shortage of NH general practitioners require innovative and adaptable models of care tailored to the organizational context. In this symposium, we will present studies from the MOQI project from the United States, which successfully reduced unnecessary hospitalizations by embedding advanced practice registered nurses (APRN) in 16 US NHs over a 6-year period. We will discuss the influence of race on multiple hospital transfers and present possible interventions to reduce transfers. Next, we will present finding from a study with MOQI APRNs that highlighted their contributions to the COVID-19 pandemic response in NHs and discuss the broader implication or infection control practices. In addition, we will present the INTERCARE project which successfully reduced unplanned hospitalizations in 11 Swiss NHs, by implementing a registered nurse with an expanded role, to compensate for the very limited access to APRNs; which is the case for many European countries. Both MOQI and INTERCARE pinpoint the importance of strategies to support the introduction of a new role in NHs. Both projects will give examples of different models of care which can be feasibly implemented to sustainably decrease unnecessary hospitalizations, in different contexts and with different resources. Finally, data from the INTERCARE study will address the issue of potentially avoidable fall-related transfers and which resources are deemed appropriate to mitigate these.

